# Retinal biomarkers for the risk of Alzheimer’s disease and frontotemporal dementia

**DOI:** 10.3389/fnagi.2024.1513302

**Published:** 2025-01-10

**Authors:** Ruihan Wang, Jiajie Cai, Yuzhu Gao, Yingying Tang, Hui Gao, Linyuan Qin, Hanlin Cai, Feng Yang, Yimeng Ren, Caimei Luo, Shiyu Feng, Hongbo Yin, Ming Zhang, Chunyan Luo, Qiyong Gong, Xiong Xiao, Qin Chen

**Affiliations:** ^1^Department of Neurology, West China Hospital of Sichuan University, Chengdu, China; ^2^Department of Epidemiology and Biostatistics, West China School of Public Health and West China Fourth Hospital, Sichuan University, Chengdu, China; ^3^Department of Ophthalmology, West China Hospital of Sichuan University, Chengdu, China; ^4^Department of Radiology, West China Hospital of Sichuan University, Chengdu, China

**Keywords:** Alzheimer’s disease, frontotemporal dementia, retina, biomarkers, optical coherence tomography

## Abstract

**Purpose:**

Differentiating between Alzheimer’s disease (AD) and frontotemporal dementia (FTD) can be challenging due to overlapping cognitive and behavioral manifestations. Evidence regarding non-invasive and early-stage biomarkers remains limited. Our aim was to identify retinal biomarkers for the risk of AD and FTD in populations without dementia and explore underlying brain structural mechanisms.

**Methods:**

We included a total of 3,0573 UK Biobank participants without dementia, ocular disorders, and diabetes who underwent baseline retinal optical coherence tomography (OCT) imaging. Cox proportional hazards models were used to estimate the associations between macular OCT parameters and the risk of AD and FTD. Mediation analysis was used to explore the underlying mechanisms affected by brain structures.

**Results:**

The mean age at recruitment was 55.27, and 46.10% of the participants were male. During a mean follow-up of 9.15 ± 2.59 years, 148 patients with AD and eight patients with FTD were identified. Reduced thickness of the ganglion cell-inner plexiform layer (GC-IPL) at baseline was associated with an increased risk of AD (HR, 1.033; 95% CI, 1.001–1.066; *P* = 0.044), while thinner retinal pigment epithelial in the inner superior subfield at baseline was associated with an elevated risk of FTD (HR, 1.409; 95% CI, 1.060–1.871; *P* = 0.018). Structurally abnormal visual pathways, including cortical and subcortical gray matter volumes, as well as white matter integrity, mediated the association between the GC-IPL thickness and AD risk.

**Conclusion:**

Our findings provide preliminary empirical support for a relationship between prodromal changes in retinal layers and a higher risk of AD or FTD, suggesting that macular OCT may serve as a non-invasive, sensitive biomarker of high-risk years before the onset of dementia.

## Introduction

Alzheimer’s disease (AD) and frontotemporal dementia (FTD) are the two most common causes of early-onset dementia (Ratnavalli et al., 2002; Lattante et al., 2015), both of which carry a substantial socioeconomic burden (Galvin et al., 2017; Livingston et al., 2020). Differentiating between AD and FTD can be challenging due to their overlapping cognitive and behavioral manifestations (Musa et al., 2020). Previous studies have highlighted the importance of using various strategies, including neuropsychological characteristics (Jiskoot et al., 2021; Maito et al., 2023), multimodal neuroimaging (Li et al., 2022; Mao et al., 2023), electroencephalogram (Si et al., 2023), cerebrospinal fluid (Mattsson-Carlgren et al., 2022; Ortner et al., 2022), and blood (Thijssen et al., 2021; Sarto et al., 2023) biomarkers, to distinguish between patients with AD and FTD. The neuropathology of AD and FTD is characterized by the accumulation of misfolded proteins, which begins several years before the onset of clinical symptoms (Benussi et al., 2022; Jack et al., 2024). The long preclinical stage provides opportunities for early disease detection and timely intervention (Jack et al., 2013; Greaves and Rohrer, 2019). However, evidence regarding non-invasive and early-stage biomarkers, even before symptom onset, to differentiate AD from FTD remains limited.

Optical coherence tomography (OCT), as a non-invasive retinal imaging tool, has provided researchers with enhanced access to detailed retinal neuronal structures (Fujimoto et al., 2000). OCT offers several advantages over brain imaging technologies or fluid biomarker testing. It is non-invasive, relatively low-cost, easy to use in primary healthcare institutions, and provides a higher spatial resolution that reveals cellular-level structures (Men et al., 2016). Currently, OCT is increasingly being applied to the study of dementing disorders for early diagnosis and disease management, including AD, FTD, and Parkinson’s disease (Douglas et al., 2021).

AD is pathologically characterized by the abnormal accumulation of amyloid beta (Aβ) and hyperphosphorylated tau proteins in the brain, which leads to subsequent neurodegeneration and progressive cognitive decline (Bloom, 2014). The pathological hallmarks of AD (amyloid beta and phosphorylated tau proteins) (Hart et al., 2016; Hart de Ruyter et al., 2023; Koronyo et al., 2023) and FTLD-tau (Kim et al., 2019; Kim et al., 2021) have been found in different sublayers of the retina in histopathological studies. Consistent with human histological (Hinton et al., 1986; Blanks et al., 1989) and postmortem (La Morgia et al., 2016) studies, previous cross-sectional studies have indicated that patients with AD dementia exhibit reduced thickness of the peripapillary retinal nerve fiber layer (RNFL) (Larrosa et al., 2014; Sánchez et al., 2018) and macular ganglion cell-inner plexiform layer (GC-IPL) (Cheung et al., 2015; Tao et al., 2019) compared to age-matched cognitively normal controls. Moreover, these retinal structural changes are reportedly associated with reduced brain volume measured by magnetic resonance imaging (MRI) (Casaletto et al., 2017; Mutlu et al., 2017; Mutlu et al., 2018b; Tao et al., 2019) Limited prospective studies with follow-up periods ranging from 12 to 27 months have investigated the longitudinal relationship between OCT parameters and the progression of AD (Mutlu et al., 2018a; Santos et al., 2018), but the findings have been inconsistent across studies. Fewer studies with small sample sizes have demonstrated that patients with FTD have thinner outer retinal thickness compared to controls (Kim et al., 2017; Kim et al., 2019) and that outer nuclear layer (ONL) thickness may be helpful in distinguishing FTLD-Tau from AD neuropathologic changes (Kim et al., 2023). Along with the easily accessible and non-invasive advantages of OCT compared to other existing imaging and fluid biomarkers, the findings above support OCT as a promising candidate for the early *in vivo* differentiation between AD and FTD. However, the limited evidence remains insufficient to identify reliable retinal biomarkers for the risk of AD and FTD due to small sample sizes and short follow-up periods.

Using a large prospective cohort from the UK Biobank, we aimed to identify retinal biomarkers for the risk of various types of dementia outcomes during long-term follow-up and further explore the underlying brain structural mechanisms linking retinal characteristics to dementia.

## Materials and methods

### Study population and design

The UK Biobank is a large prospective study consisting of more than 500,000 participants aged 40–69, recruited from one of 22 centers located across Scotland, England, and Wales. The recruitment was conducted between 2006 and 2010, with written informed consent provided by all participants. The details of the UK Biobank study design and population have been described previously (Sudlow et al., 2015).

**Figure 1:**
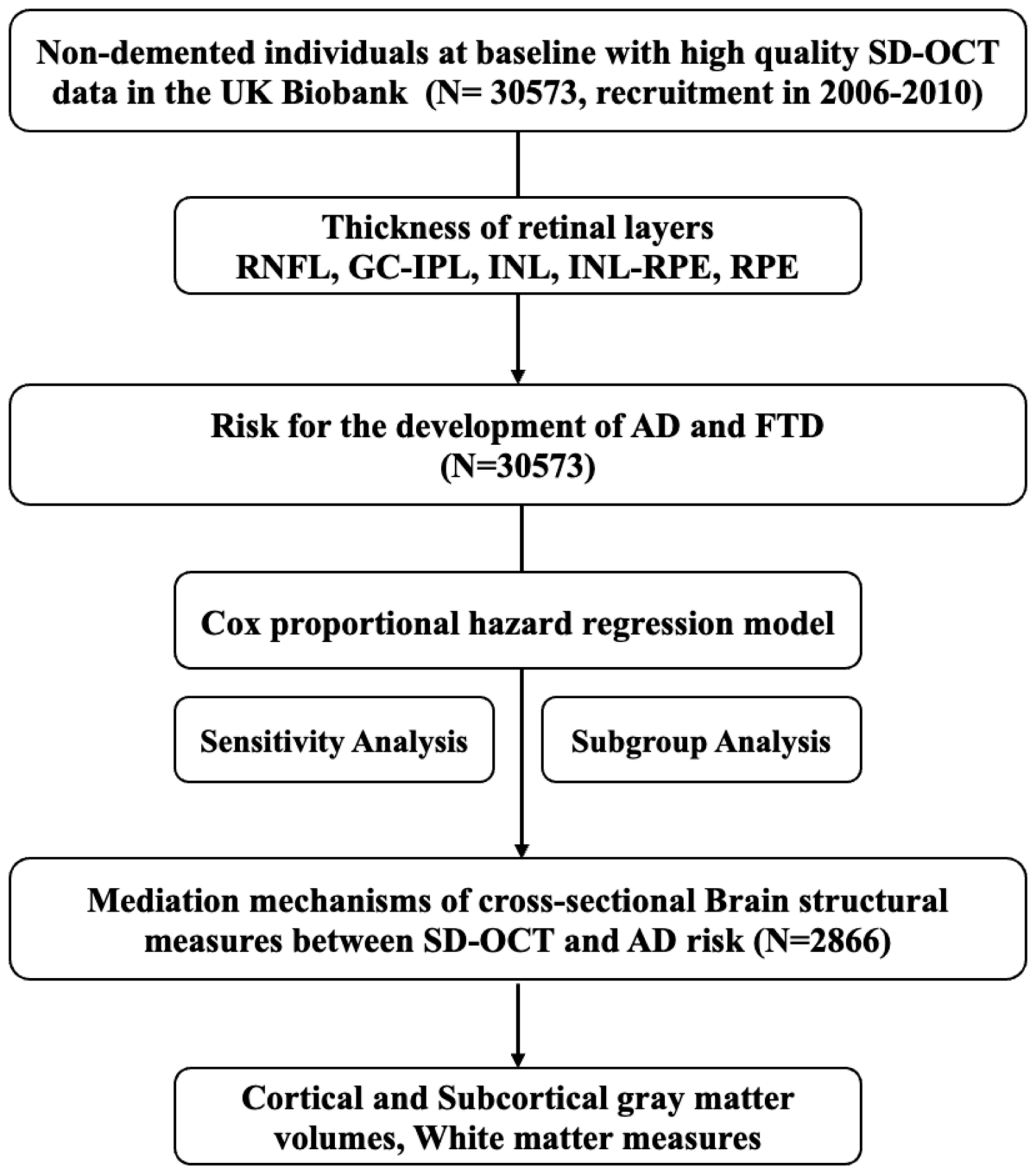
Study design and workflow. SD-OCT, Spectral-domain Optical Coherence Tomography; RNFL, retinal nerve fiber layer; GC-IPL, ganglion cell-inner plexiform layer; INL, inner nuclear layer; INL-RPE, inner nuclear layer-retinal pigment epithelial; RPE, retinal pigment epithelium; ACD, all-cause dementia; AD, Alzheimer’s disease; FTD, frontotemporal dementia.

This prospective cohort study included individuals with high-quality macular spectral-domain optical coherence tomography (SD-OCT) images, without prevalent dementia, ocular disorders, or diabetes, for the analysis of OCT parameters and the risk of incident dementia. Furthermore, a subgroup of those with additional brain MRI data were eligible for the mediation analysis. This study was conducted using the UK Biobank Resource under Application Number 98130, and the study design is presented in Figure 1.

### Ophthalmic examination and SD-OCT

Ophthalmic examinations, including visual acuity, autorefraction/keratometry (Tomey RC5000; Erlangen-Tennenlohe), Goldmann-corrected intraocular pressure (IOP), and cornea-corrected IOP (Ocular Response Analyzer; Reichert), were performed at six centers during 2009–2010. SD-OCT imaging was performed using the Topcon 3D OCT-1000 Mk2 (Topcon, Tokyo, Japan). Image acquisition was performed under mesopic conditions, without pupillary dilation, using the three-dimensional macular volume scan (512 horizontal A scans per B scan, 128 B scans in a 6 × 6 mm2 raster pattern). The OCT protocol was described in detail by Ko et al. (2017) and Patel et al. (2016) and is compliant with the APOSTEL guidelines.

From the initial sample of 5,02,359 participants, our study included those with high-quality spectral-domain optical coherence tomography imaging data, according to the OSCAR-IB criteria (Cruz-Herranz et al., 2016). Image quality scores and segmentation indicators provided by the Topcon Advanced Boundary Segmentation software were used for quality control. The exclusion criteria for all eligible individuals were as follows: (1) an image quality score < 45, poor centration certainty, or poor segmentation certainty (the poorest 20% of images were excluded based on each of the segmentation indicators); (2) participants with ocular-related comorbidities: refractive error > 6D or < -6D, best-corrected visual acuity > 0.1 logMAR, IOP ≥ 22 mmHg or ≤ 5 mmHg, or a history of ocular diseases (i.e., recent eye surgery in the last 4 weeks, corneal graft, eye trauma, macular degeneration, retinal detachment, retinal artery/vein occlusion, cataract, retinal problem, or glaucoma) or diabetes; and (3) participants with cognitive impairment or dementia, identified by self-reporting or diagnosed by hospital records prior to the date of baseline assessment. Finally, if both eyes of an individual were eligible for inclusion in this analysis, one eye was chosen randomly. The selection procedure is described in Supplementary material 1. The Topcon Advanced Boundary Segmentation (TABS) algorithm was used to automatically segment the inner and outer retinal boundaries, as well as the retinal sublayers (Keane et al., 2016). The segmented boundaries include the internal limiting membrane (ILM), nerve fiber layer (NFL), ganglion cell layer (GCL), inner plexiform layer (IPL), inner nuclear layer (INL), external limiting membrane (ELM), photoreceptor inner segment/outer segment (ISOS) junction, retinal pigment epithelium (RPE), and Bruch’s membrane (BM). The overall retinal thickness in the macular region, as well as the inner (RNFL, GC-IPL, INL) and outer (INL-RPE, RPE) retinal sublayers, was analyzed. To evaluate the structural integrity of the photoreceptor cells, we selected the INL-ELM region, which includes the outer nuclear layer (ONL), primarily composed of photoreceptor cell nuclei, and the outer plexiform layer (OPL), which contains their axons. Furthermore, the ELM-ISOS and ISOS-RPE regions, which encompass the inner and outer segments of photoreceptors, including their mitochondria, were also included to provide a more comprehensive assessment of potential pathological changes. The nine subfields of the RPE layer were defined by the Early treatment diabetic retinopathy study circle (Early Treatment Diabetic Retinopathy Study Research Group, 1991).

### Incident dementia

We identified patients with dementia (all-cause dementia [ACD], Alzheimer’s disease [AD], vascular dementia [VaD], and frontotemporal dementia [FTD]) based on a primary/secondary diagnosis using the International Classification of Diseases (ICD) coding system (detailed in Appendix 1) from the UK Biobank inpatient hospital records in the Hospital Episode Statistics database, the Scottish Morbidity Record, and the Patient Episode Database. Additional patients with dementia and MCI were defined based on underlying/contributory causes of death according to the death register data (ICD codes).

### MRI data management

UK Biobank brain MRI data with a resolution of 1 × 1 × 1 mm were collected using a standard Siemens Skyra 3 T (Siemens Healthcare, Erlangen, Germany) scanner with a 32-channel head coil, and the sequence parameters have been reported previously (Alfaro-Almagro et al., 2018). Cross-sectional MRI data from individuals who underwent a baseline MRI scan in 2014 were included to determine brain structural measures. T1-weighted images were obtained with a 3D magnetization-prepared rapid gradient-echo (MPRAGE) sequence as the scanning protocol. We used global and regional brain imaging-derived phenotypes (IDPs) provided by the UK Biobank brain imaging team. FreeSurfer was used to analyze T1 images, employing surface templates (Desikan–Killiany parcellation) to obtain IDPs available in the UKB category of FreeSurfer aparc (ID = 192), referring to the cortical volume of 34 bilateral cortical regions. The FMRIB’s Automated Segmentation Tool (FAST) of FMRIB Software Library (FSL) (Zhang et al., 2001) was also applied for cortical gray matter segmentation to generate 139 IDPs in the UKB category of 1,101. The FSL’s FIRST (Patenaude et al., 2011) was implemented to extract the volume of seven bilateral key subcortical structures, including the accumbens, amygdala, thalamus, hippocampus, pallidum, caudate, and putamen, in the UKB category of 1,102. Diffusion MRI parameters, including fractional anisotropy (FA) and mean diffusivity (MD), of 27 white matter tracts defined by AutoPtx (de Groot et al., 2013) were analyzed using the FSL. Regional values were averaged across the left/right hemispheres.

### Statistical analysis

**Figure 2:**
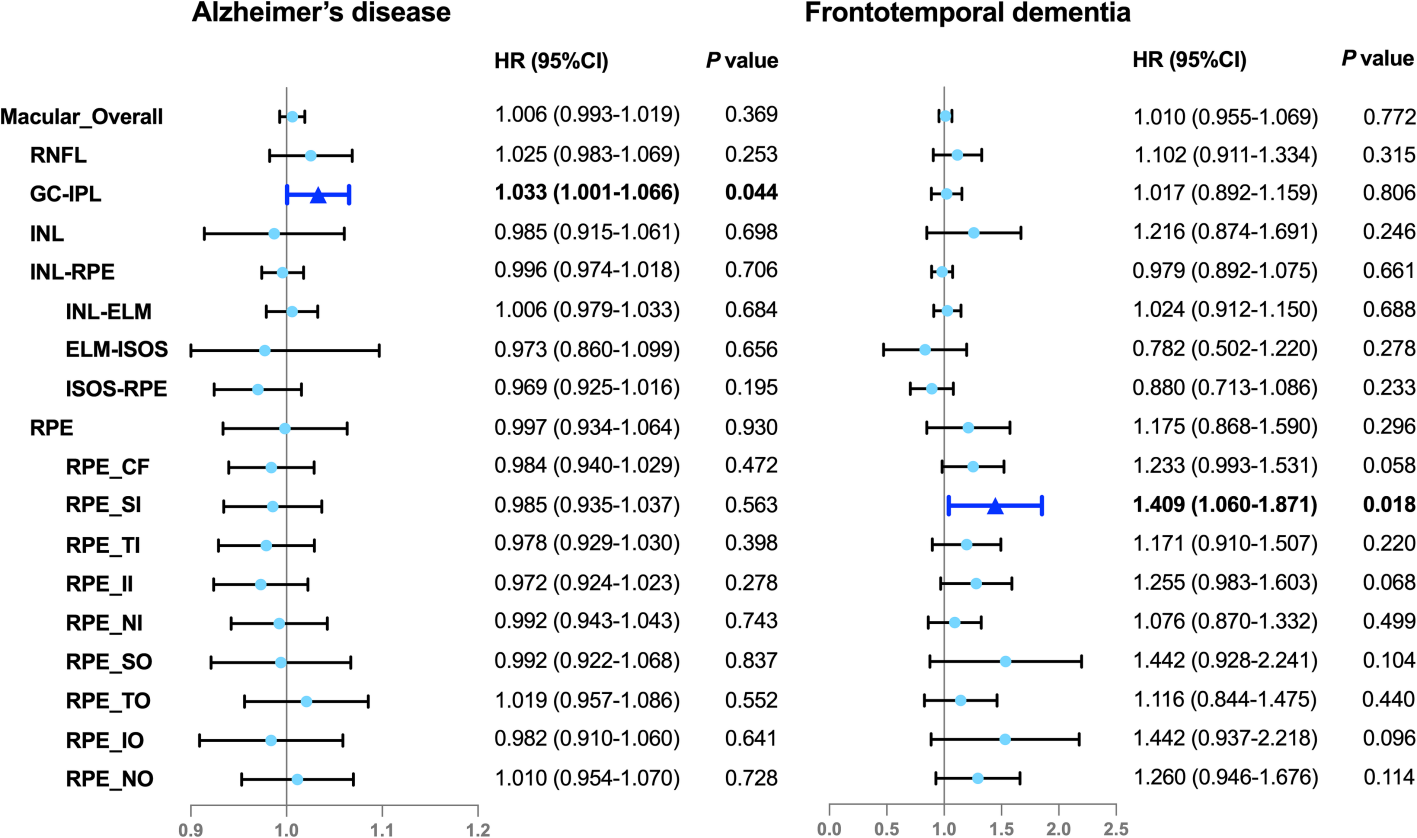
Associations between the OCT parameters and incident dementia. Illustration of the Cox proportional hazards analyses. The solid color box represents the statistically significant associations (*P* < 0.05), whereas the lighter color box indicates null associations (*P* > 0.05). The model was adjusted for sociodemographic factors (age, sex, ethnicity, education, and the Townsend deprivation index), smoking status, disease-related risk factors (hypertension, hyperlipidemia, cardiovascular disease, and *APOE* ε4 status), height, and ocular factors (refractive error, intraocular pressure). HR, hazard ratio; 95% CI, 95% confidence interval; RNFL, retinal nerve fiber layer; GC-IPL, ganglion cell-inner plexiform layer; INL, inner nuclear layer; INL-RPE, inner nuclear layer-retinal pigment epithelial; INL-ELM, inner nuclear layer-external limiting membrane; ELM-ISOS, external limiting membrane-inner segment outer segment; ISOS-RPE, inner segment outer segment-retinal pigment epithelium; RPE, retinal pigment epithelium; CF, central subfield; SI, inner superior subfield; TI, inner temporal subfield; II, inner inferior subfield; NI, inner nasal subfield; SO, outer superior subfield; TO, outer temporal subfield; IO, outer inferior subfield; NO, outer nasal subfield.

We characterized the population using means (standard deviations) and frequencies (proportions) for continuous and categorical variables, respectively. Cox proportional hazards models were used to assess the associations between the OCT parameters and the risk of developing various dementia subtypes. No significant violation of the proportional hazard assumptions was detected using the Schoenfeld residuals method (Supplementary material 3; Figure 2). Information about the missing data is available in Appendix 2. In addition, we used restricted cubic spline curves with three nodes (10th, 50th, and 90th percentiles) to examine the non-linearity of the associations between SD-OCT and the risk of dementia. The Cox proportional hazards analyses were conducted using three models. Model 1 was not adjusted for any covariates. In Model 2, we adjusted for sociodemographic factors (age, sex, ethnicity, education, and the Townsend deprivation index) and smoking status. In Model 3, we further adjusted for disease-related risk factors (hypertension, hyperlipidemia, cardiovascular disease, and *APOE* ε4 status), height, and ocular factors (refractive error, IOP).

To further examine the role of the brain MRI structure in the relationship between the OCT parameters and the development of dementia, mediation analyses were applied in the subsequent steps. We first screened the OCT parameters that might be associated with dementia risk using the Cox proportional hazards model. Next, we developed multiple linear regression models for the OCT parameters with brain MRI structures and used the Cox proportional hazards model to assess the association between the MRI structures and dementia outcomes. MRI parameters for mediation analysis were obtained based on statistically significant results, and we used a Bootstrap mediation model (1,000 replicates) to estimate the extent to which MRI parameters can explain the association between OCT parameters and dementia risk, along with the associated 95% confidence interval. To control for potential confounders, we adjusted for sociodemographic factors (age, sex, ethnicity, education, and the Townsend deprivation index), smoking status, disease-related risk factors (hypertension, hyperlipidemia, cardiovascular disease, and *APOE* ε4 status), height, and ocular factors (refractive errors, and IOP). Then, the indirect effect (β_IE_) and the mediation proportion were reported (mediation proportion = natural indirect effect/ [natural direct effect + natural indirect effect]).

To examine potential effect modifiers, we performed stratified analyses of the predefined subgroups, including age (≤ 55 and > 55 years), sex (male or female), and the presence of cardiovascular disease (CVD) at baseline. We also performed two sensitivity analyses to examine the robustness of our findings. First, we excluded the occurrence of specific dementia outcome events in the first 5 years of the follow-up to rule out reverse causation. Second, we excluded populations with the presence of the *APOE* ε4 allele to explore the risk of dementia in populations without the genetic effect. Age- and sex-matched cohort analyses were conducted in the participants who developed AD or FTD and those without incident dementia to further estimate the reliability of our findings.

FDR correction was used to alleviate the problem of multiple testing of various MRI parameters, with adjusted *P*-values reported. R software (version 4.3.1) was used for statistical analysis and data processing. A two-sided *P*-value of < 0.05 was considered statistically significant.

## Results

### Baseline characteristics

The characteristics of the participants stratified by incident dementia are summarized in Table 1. Of the 30,573 participants without prevalent dementia who had high-quality OCT imaging data at baseline, 295 developed ACD during an average of 9.15 ± 2.59 years of follow-up, comprising 148 patients with AD, eight patients with FTD, and 42 patients with VaD. Compared to the group without dementia, the group with dementia was more likely to be older, less educated, smokers, *APOE* ε4 carriers, diagnosed with hypertension, hyperlipidemia, and CVD, and to have higher refractive error and worse visual acuity. The participants who developed dementia during the follow-up had significantly lower baseline retinal thickness in the macular area, including overall retinal thickness and the thickness of the retinal sublayers (RNFL, GC-IPL, and RPE), compared to those who did not develop dementia (Table 1).

**Table 1:** Baseline characteristics of the study participants by incident dementia status.

Characteristics	All participants (*N* = 30,573)	No incident dementia (*N* = 30,278)	Incident dementia (*N* = 295)	*P*-value
Age	55.27 (8.19)	55.18 (8.17)	63.82 (4.94)	<0.001***
Male (%)	14,081 (46.1%)	13,920(46.0%)	161(54.6%)	0.387
Ethnicity, No. (%)				0.475‡
White	27,964 (91.9%)	27,684 (91.6%)	280 (95.2%)	
Mixed/other ethnic group	714 (2.3%)	709 (2.3%)	5 (1.7%)	
Asian/Indian	785 (2.6%)	780 (2.6%)	5 (1.7%)	
Black	825 (2.7%)	821 (2.7%)	4 (1.4%)	
Chinese	92 (0.3%)	92 (0.3%)	0	
Education, No. (%)				0.003*
Low	1,627 (6.1%)	1,603 (6.1%)	24 (11.5%)	
Moderate	9,186 (34.4%)	9,112 (34.4%)	74 (35.6%)	
High	15,863 (59.5%)	15,753 (59.5%)	110 (52.9%)	
Laterality = right eye	13,981 (45.7)	13,848 (45.7)	133 (45.1)	0.869
BMI, kg/m^2^	27.08 (4.56)	27.08 (4.56)	27.31 (4.66)	0.387
Smoking status (%)				<0.001***
Never	16,947 (55.7%)	16,821 (55.9%)	126 (43.4%)	
Previous	10,466 (34.4%)	10,328 (34.3%)	138 (47.6%)	
Current	2,990 (9.8%)	2,964 (9.8%)	26 (9.0%)	
Alcohol status (%)				0.670
Never	1,256 (4.1)	1,244 (4.1)	12 (4.1)	
Previous	966 (3.2)	954 (3.2)	12 (4.1)	
Current	28,241 (92.6)	27,971 (92.6)	270 (91.8)	
Height (cm)	169.11 (9.23)	169.12 (9.23)	168.34 (9.54)	0.166
Townsend deprivation index	−1.13 (2.93)	−1.13 (2.93)	−1.31 (2.84)	0.276
Visual acuity, logMAR	−0.07 (0.09)	−0.07 (0.09)	−0.04 (0.08)	<0.001***
Refractive error	−0.01 (1.86)	−0.02 (1.86)	0.63 (1.86)	<0.001***
Intraocular pressure	15.15 (3.10)	15.15 (3.1)	15.43 (2.92)	0.106
Hypertension (%)	7,869 (25.7)	7,689 (25.4)	180 (61.0)	<0.001***
Hyperlipidemia (%)	4,311 (14.1)	4,191 (13.8)	120 (40.7)	<0.001***
Cardiovascular disease (%)	3,378 (11.0)	3,274 (10.8)	104 (35.3)	<0.001***
Ischemic heart disease (%)	2,869 (9.4)	2,786 (9.2)	83 (28.1)	<0.001***
Stroke (%)	600 (2.0)	564 (1.9)	36 (12.2)	<0.001***
*APOE* ε4 (%)				<0.001***
0 allele	17,991 (71.2)	17,895 (71.5)	96 (40.9)	
1 allele	6,634 (26.3)	6,528 (26.1)	106 (45.1)	
2 allele	628 (2.5)	595 (2.4)	33 (14.0)	
Overall macular thickness	278.32 (12.99)	278.35 (12.99)	275.83 (12.67)	<0.001***
RNFL	28.47 (4.11)	28.48 (4.11)	27.55 (3.99)	<0.001***
GC-IPL	74.98 (5.43)	75.00 (5.43)	73.47 (5.44)	<0.001***
INL	32.68 (2.28)	32.68 (2.28)	32.59 (2.32)	0.498
INL-RPE	142.18 (7.58)	142.18 (7.58)	142.2 (7.19)	0.952
RPE	25.39 (2.91)	25.39 (2.91)	25.03 (2.43)	0.012*

Data are presented as mean (SD) or *n* (%). The *P*-values were calculated using the t-test for the continuous variables or the chi-square test for the categorical variables.

^‡^Use Fisher’s Exact Test. ***indicates a *P*-value < 0.001. *indicates a *P*-value < 0.05.

BMI, body mass index; APOE ε4, Apolipoprotein E4; RNFL, retinal nerve fiber layer; GC-IPL, ganglion cell-inner plexiform layer; INL, inner nuclear layer; INL-RPE, inner nuclear layer-retinal pigment epithelial; RPE, retinal pigment epithelium.

### Associations between the OCT parameters and dementia risk

The analyses with hazard ratios (HRs) and 95% confidence intervals (95% CIs) is shown in Figure 2. All OCT parameters were treated as continuous variables. The Cox proportional hazards models showed that the thinner RPE in the inner superior subfield was associated with a 40.9% increased risk of FTD (HR, 1.409; 95% CI, 1.060–1.871; *P* = 0.018) and the lesser average GC-IPL thickness was associated with a 3.3% higher risk of AD (HR, 1.033; 95% CI, 1.001–1.066; *P* = 0.044) (Figure 2). Similar findings were observed in the subsets of the cohort comprising the participants with incident AD or FTD and the age- and sex-matched control individuals without incident dementia (Supplementary material 6). No evidence of non-linearity was indicated across the models (Supplementary material 3; Figure 1). However, we did not find any associations between any OCT parameters and ACD or VaD.

In the age-specific models, the thinning of the GC-IPL was significantly associated with an increased risk of AD in the individuals aged over 55 years, while the thickness of the RPE in the inner superior subfield showed an inverse association with FTD in those aged ≤ 55 years (Supplementary material 4). In the stratified analyses by sex and CVD status, the male participants and individuals with CVD exhibited a more significant association between the GC-IPL thickness and AD risk, as well as between the RPE thickness in the inner superior subfield and FTD risk (Supplementary material 4). When restricting the analytical samples based on the follow-up period longer than 5 years, there was no significant difference between the thickness of the RPE in the inner superior subfield and FTD (Supplementary material 5). In addition, in the participants without the *APOE* ε4 allele, there was a slight association between the thickness of the GC-IPL and the risk of AD (Supplementary material 5).

### Potential neuroimaging mechanisms underlying the association between the GC-IPL and AD

**Figure 3:**
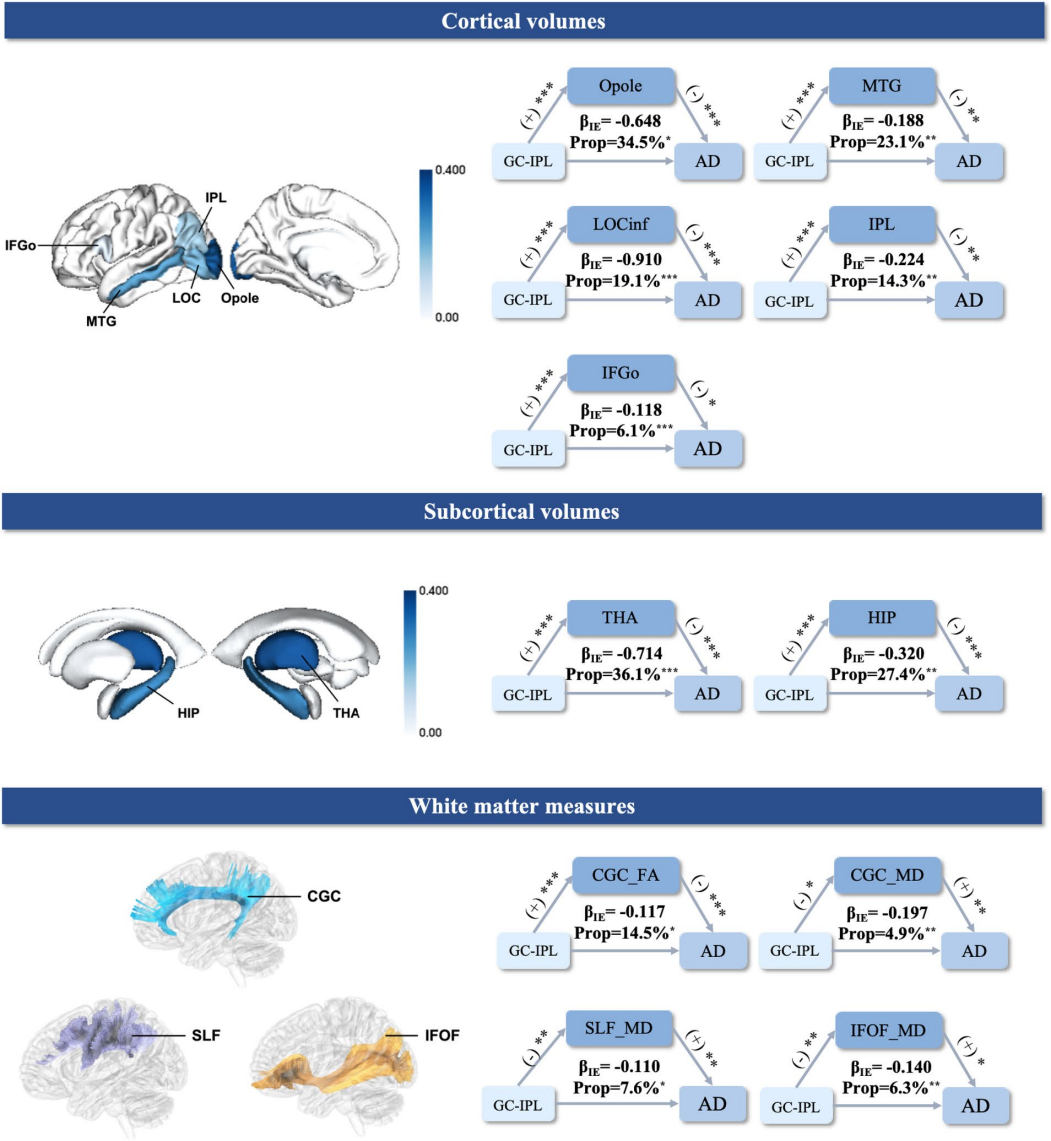
Mediation analysis of the relationships between the GC-IPL, brain structural measures, and AD. The mediation results (top) between the GC-IPL thickness, cortical gray matter volume, and risk of AD. (middle) The mediation results between the GC-IPL thickness, subcortical gray matter volume, and risk of AD. (bottom) The mediation results between the GC-IPL thickness, white matter measures, and risk of AD. (+) indicates a positive association. (−) indicates a negative association. β_IE_ represents the indirect effects of the GC-IPL on AD. ***indicates a *P*-value <0.001. **indicates a *P*-value <0.01. *indicates a *P*-value <0.05. The color bars reflect the value of the mediation proportion. GC-IPL, ganglion cell-inner plexiform layer; AD, Alzheimer’s disease; IFGo, Inferior Frontal Gyrus, pars opercularis; LOCinf, Lateral Occipital Cortex, inferior division; Opole, Occipital Pole; IPL, Inferior Parietal Lobule; MTG, Middle Temporal Gyrus; THA, thalamus; HIP, hippocampus; CGC, cingulate gyrus part of cingulum; IFOF, inferior fronto-occipital fasciculus; SLF, superior longitudinal fasciculus; FA, fractional anisotropy; MD, mean diffusivity; Prop: mediation proportion.

The average thickness of the GC-IPL was selected as an OCT parameter as it showed a significant association between the retinal structures and AD. Neuroimaging mechanisms were investigated by examining whether the GC-IPL-related association with AD risk was mediated by specific brain structural biomarkers. The mediation analyses suggested that reduced regional cortical and subcortical gray matter volumes were statistically significant in mediating the associations between the thinner GC-IPL and increased risk of AD (occipital pole 34.5%, middle temporal gyrus 23.1%, lateral occipital cortex inferior division 19.1%, inferior parietal lobule 14.3%, inferior frontal gyrus pars opercularis 6.1%, thalamus 36.1%, and hippocampus 27.4%). As for the white matter measures, lower FA in the cingulate gyrus part of the cingulum (9.3%), as well as higher MD in the superior longitudinal fasciculus (SLF, 7.6%), inferior fronto-occipital fasciculus (IFOF, 6.3%) and the cingulate gyrus part of the cingulum (CGC, 4.9%), might have played a role in the association between the thickness of the GC-IPL and increased AD risk (Figure 3).

## Discussion

The current study provides evidence of the longitudinal association between OCT parameters and the risk of incident dementia based on a large cohort of 30,573 UK Biobank participants. We found that the thinner GC-IPL was associated with an increased risk of Alzheimer’s disease, while the thinner RPE in the inner superior subfield was associated with an increased risk of FTD. In addition, reduced thickness of the retinal layers may potentially contribute to AD by decreasing brain volumes associated with the visual pathway. Our findings strongly support that early changes in the retinal layers may serve as presymptomatic biomarkers years before dementia onset.

In this study, the OCT parameters were significantly associated with AD and FTD, with tau pathology potentially playing a contributory role (Samudra et al., 2023). However, no significant association was found between any OCT parameters and ACD or VaD. These findings suggest that retinal structural changes measurable by OCT may be affected by pathophysiological mechanisms related to tauopathies rather than dementia itself.

We found that the thinner GC-IPL was associated with an increased risk of incident AD, which is consistent with the findings of limited prospective studies (Mutlu et al., 2018a; Santos et al., 2018). As demonstrated in cross-sectional studies, the GC-IPL, one of the inner layers of the retina, exhibited pronounced pathology and thinning in AD during the autopsy (Hart et al., 2016; Koronyo et al., 2023). It was also thinner in the patients with AD and mild cognitive impairment (MCI) compared to the controls (Cheung et al., 2015; Tao et al., 2019) and correlated with clinical disease severity (Kim and Kang, 2019; Liu et al., 2019). Some studies have proposed that the impairment of the cell bodies and dendrites of retinal ganglion cells (i.e., GC-IPL) may be connected with several neurotoxic pathways related to AD pathology, such as increased inflammatory pathways, oxidative stress, and vascular alterations (Wang and Mao, 2021; Koronyo et al., 2023), which may explain the thinning of the GC-IPL in the preclinical stage of AD in our study. However, we did not find any significant association between the axon layer of the retinal ganglion cells (macular RNFL) and the incidence of AD. As a previous study (Santos et al., 2018) was based on a relatively short follow-up period, the inconsistency may suggest that damage to the cell bodies of retinal ganglion cells precedes damage to the axons in the disease progression of AD (Cheung et al., 2015; Ito et al., 2020). Our study further supports that alterations in the cell bodies of retinal ganglion cells are a sensitive retinal biomarker for AD.

Our results indicated that the cortical and subcortical regions involved in the visual pathways mainly mediate the association between the thickness of the GC-IPL and the risk of incident AD. Cross-sectional studies have demonstrated that lower GC-IPL thickness is significantly associated with reduced volume in brain regions mainly located in the occipital and temporal lobes among cognitively healthy individuals and patients with AD (Ong et al., 2015; Tao et al., 2019), even in the preclinical stage of AD with normal cognition (Casaletto et al., 2017; Mutlu et al., 2017) and Aβ deposition (Byun et al., 2021). However, no study has suggested possible associations between the GC-IPL and AD via the mechanism of brain structural alterations. The visual pathways emphasized in mediating this relationship in our study were demonstrated to begin in the retina, travel through the optic tract to the LGN in the thalamus, terminate at the primary visual cortex (V1), and then reach the extrastriate areas (V2-V5) via the ventral and dorsal streams (Joukal, 2017). As key components of the visual pathways, the occipital pole encompassing V1-V4 (Jones et al., 2020) and the thalamus including the LGN were found to have a maximal mediation proportion of over 30%. The regions including the middle temporal area, inferior parietal lobule, inferior frontal gyrus, and lateral occipital cortex were shown to have a potential mediating role in our study as well. These regions were demonstrated to be involved in the dorsal stream for the perception of movement and space (Rizzolatti and Matelli, 2003) and the ventral visual pathway supporting object recognition (Grill-Spector et al., 2001), respectively. In addition, consistent with a cross-sectional study showing the relationship between retinal structure changes and hippocampal atrophy in patients with AD and MCI (Zhao et al., 2020), our study further provides evidence that the hippocampus may serve as a link between the thickness of the GC-IPL and AD. It may be because the hippocampus is not only essential for the memory system (Fortin et al., 2002) but also implicated in spatial perception, eye movements, and scene construction as a visual area (Turk-Browne, 2019).

We demonstrated that the lower integrity (lower FA and/or higher MD) of the visual-related white matter tracts including, the CGC, IFOF, and SLF, mediated the association between the thickness of the GC-IPL and AD. Previous studies have found that the thinner GC-IPL is associated with lower FA and higher MD in the optic radiation of both normal elders (Mutlu et al., 2018b) and patients with AD (Hao et al., 2023). Consistently, our study showed the IFOF, which completely covers the superior optic radiation along its entire course (Sarubbo et al., 2015), may mediate the association between the thickness of the GC-IPL and AD. Furthermore, our study also suggested the potential involvement of lower white matter integrity in the cingulum and SLF, which has been observed in cross-sectional studies of patients with AD (Zhao et al., 2021; Xiao et al., 2022) and has been suggested to serve as a significant predictor of worse visuomotor performance in *APOE* ε4 carriers (Rogojin et al., 2022). Our findings further suggested that white matter degeneration may be part of the brain’s structural mechanism between the thickness of the GC-IPL and AD.

Our study demonstrated that the thinning of the outer retina was associated with an increased risk of FTD, which is consistent with the findings of previous cross-sectional studies (Kim et al., 2017; Kim et al., 2023). The outer retina is composed of the photoreceptor layers through to the choroid, including the ONL, ellipsoid zone (EZ), and RPE. However, previous research has primarily focused on the thinning of the ONL and EZ in patients with symptomatic FTD (Kim et al., 2017; Kim et al., 2021). In our study, the thinning of the RPE in the inner superior subfield was observed at an earlier stage of FTD. We suppose that the thinning of the RPE may lead to secondary metabolic disturbances in photoreceptor cells, resulting in the loss of the ONL, as the ONL contains the cell bodies of photoreceptor cells and the RPE plays a crucial role in supporting the function and survival of these cells (Wang et al., 2023). Future studies could provide more details on the sequence of retinal changes, including the RPE and ONL, in FTD.

The subgroup analyses suggested age and sex differences in the retinal biomarkers and dementia in the current study. A significant association was observed between the GC-IPL and AD in the older group, while an association between the RPE and FTD was found in the younger group. The finding may be due to the age-specific incidence and prevalence of AD, which increases markedly after the age of 65 (Alzheimer's Association Report, 2023), while approximately 60% of people with FTD are between 45 and 64 years old (Knopman and Roberts, 2011). In addition, we reported that the negative association between the thinning GC-IPL and RPE in the inner superior subfield and AD and FTD, respectively, was more significant in the male patients when compared to the female patients, which has not been found in other OCT studies. However, previous studies have highlighted the role of postmenopausal drops in estrogen levels in increasing the incidence of ocular symptoms and ocular diseases (Peck et al., 2017). Moreover, RNFL thickness was found to be lower with increasing age and hormonal decline (Deschênes et al., 2010; Fathy et al., 2022). This suggests that estrogen may be a protective factor for retinal structures and could partly explain the sex differences in this association. Future studies should place greater emphasis on the sex differences in retinal structures and dementia. In addition, a more significant association between the GC-IPL and AD was found in the individuals with CVD in our study. Vascular pathology has been identified as the most common co-pathology in AD (Robinson et al., 2021) and could accelerate AD pathology and cognitive decline (Agrawal and Schneider, 2022).

The sensitivity analyses showed that the follow-up period and *APOE* ε4 status might have affected the findings between the retinal biomarkers and the risk of incident dementia. The relationship between the RPE and FTD was not significant when excluding the participants with less than 5 years of follow-up, which may be due to the small sample size of the participants with FTD. *APOE* ε4 status may influence the association between the GC-IPL and AD, as indicated in our study. Similarly, previous studies also suggested that retinal abnormalities were found in cognitively unimpaired *APOE* ε4 carriers compared to non-carriers (Sheriff et al., 2023). Future studies should focus on the time span of retinal structural alterations, various types of dementia, and other genetic and co-pathological factors in this association.

The advantages of this study include a large sample size, prospective design, and long-term follow-up. However, the current study also has some limitations. First, the dementia outcomes were ascertained based on the ICD codes derived from electronically linked data, but the status of amyloid-tau-neurodegeneration (ATN) (Jack et al., 2018), a biological classification system for AD diagnosis, was not included. This might have resulted in the potential misclassification of the participants. Secondly, the participants in the UK Biobank cohort appeared to be healthier than the general population (Hanlon et al., 2022), which might have led to a relatively small number of participants with dementia during the follow-up (especially as only eight participants developed FTD) and an even smaller sample size with brain MRI. Therefore, the statistical power of the association between the OCT parameters and FTD might have been affected and the mediation analysis of the RPE, brain structures, and FTD could not be conducted. Thirdly, this study focused on macular imaging using SD-OCT, and the peripheral RNFL, which might be associated with AD, as suggested in previous studies (Shi et al., 2014; Mutlu et al., 2018a), was not included. Finally, the representativeness of the study is limited as the participants were predominantly white. The findings of this study may not be applicable to other ethnic groups. Future research should be conducted in more diverse cohorts.

In conclusion, this study provides evidence for the longitudinal association between macular OCT parameters and various types of dementia in the general population. Given that OCT is widely available, relatively affordable compared to other neuroimaging techniques, and produces rapid, high-resolution images, it might be a practical option for widespread screening, especially when compared to traditional cognitive tests, which can be time-consuming and stressful for patients. Collectively, our study supports OCT as a safe, non-invasive screening tool for early-stage AD and FTD.

## Data Availability

Publicly available datasets were analyzed in this study. This data can be found at: https://www.ukbiobank.ac.uk/.
