# Supplementary material for: Retinal biomarkers for the risk of Alzheimer’s disease and frontotemporal dementia
Source: Front Aging Neurosci. 2025 Jan 10;16:1513302. doi: 10.3389/fnagi.2024.1513302 (PMC11759267; doi:10.3389/fnagi.2024.1513302)
Supplement: Supplementary file 1 [file Data_Sheet_1.docx]

**Supplementary Materials**

Retinal biomarkers for the risk of Alzheimer’s disease and frontotemporal dementia

Supplementary Material 1 - Inclusion/Exclusion Criteria for Macular SD-OCT 2

SM 1 Figure 1 - Workflow of Macular SD-OCT Inclusion/Exclusion 2

SM 1 Table 1 - UK Biobank codes for Macular SD-OCT Inclusion/Exclusion 3

Supplementary Material 2 - Primary analyses 5

SM 2 Table 1 - Primary Cox regression models of Alzheimer’s disease 5

SM 2 Table 2 - Primary Cox regression models of Frontotemporal dementia 6

Supplementary Material 3 - Nonlinear test and violation of the proportional hazard assumptions 7

SM 3 Figure 1 - Restricted cubic spline curves 7

SM 3 Figure 2 - Schoenfeld's residuals test for Cox proportional hazard regression models 8

Supplementary Material 4 - Subgroup analyses 9

SM 4 Table 1 - Association Between GC-IPL and Alzheimer’s disease in subgroups 9

SM 4 Table 2 - Association Between RPE_SI and Frontotemporal dementia in subgroups 10

Supplementary Material 5 - Sensitivity analyses 11

SM 5 Table 1 - Follow-up-restricted models 11

SM 5 Table 2 - *APOE* ε4-restricted models 12

Supplementary Material 6 – Age- and sex- matched cohort analyses 11

SM 6 Table 1 - Baseline characteristics of study participants by incident AD status 11

SM 6 Table 2 - Baseline characteristics of study participants by incident FTD status 12

SM 6 Figure 1 - Associations of OCT parameters and incident AD/FTD 12

Appendix 1 - UK Biobank codes for dementia diagnosis and classification 13

Appendix 2 - Information of missing data 14

**Supplementary Material 1. Inclusion/Exclusion Criteria for Macular SD-OCT**

**SM 1 Figure 1 - Workflow of Macular SD-OCT Inclusion/Exclusion**


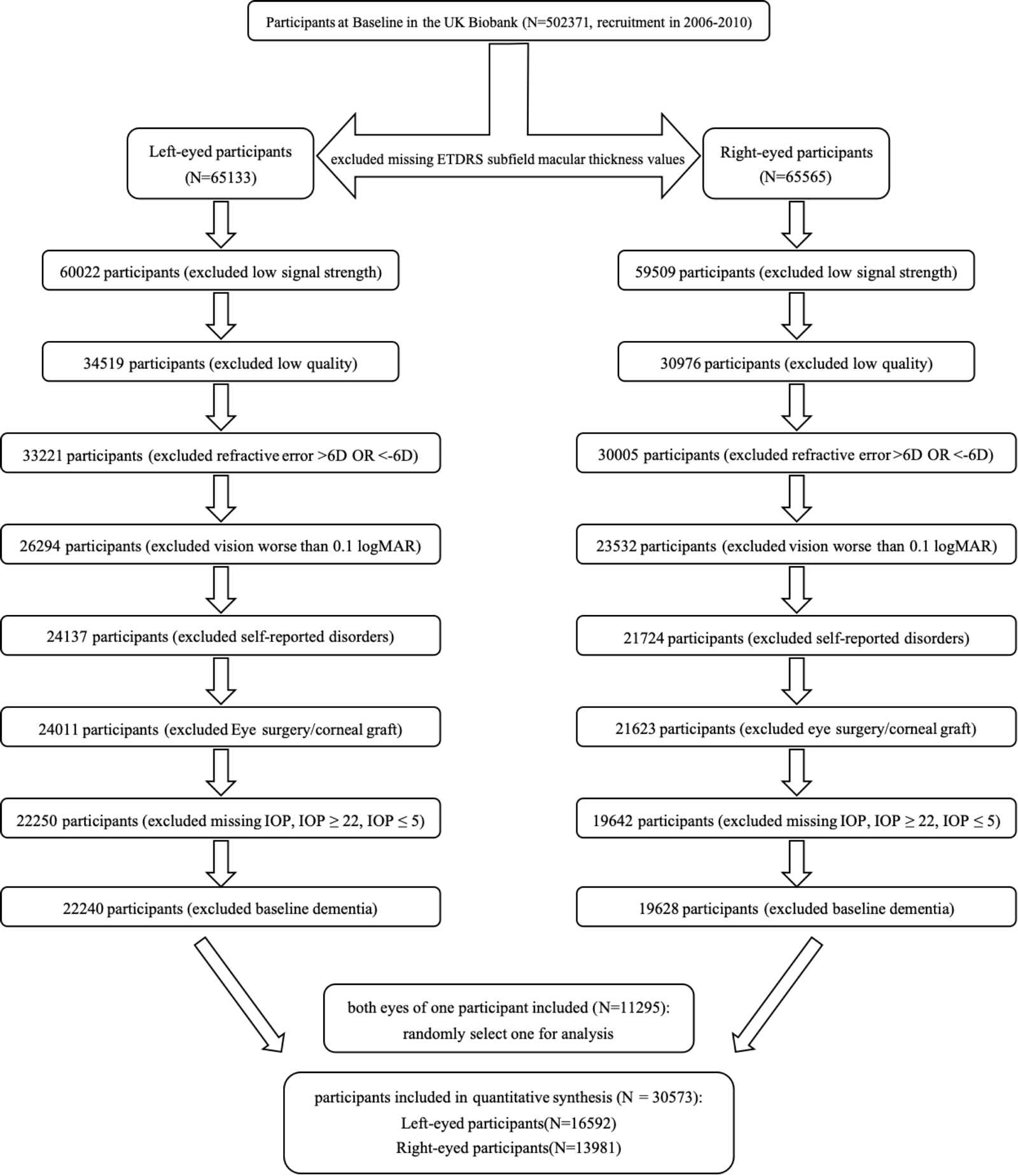


**SM 1 Table 1 - UK Biobank codes for Macular SD-OCT Inclusion/Exclusion**

| Exclusion procedure | Exclude details | Full name in UK Biobank data dictionary | Field ID (Coding) |
| --- | --- | --- | --- |
| Excluded missing ETDRS subfield macular thickness values | The left-eyed dataset excludes the participants where the right parameter is the missing value | Macular thickness at the central subfield (left) | 27802 |
|  |  | Macular thickness at the inner superior subfield (left) | 27808 |
|  |  | Macular thickness at the inner temporal subfield (left) | 27810 |
|  |  | Macular thickness at the inner inferior subfield (left) | 27804 |
|  |  | Macular thickness at the inner nasal subfield (left) | 27806 |
|  |  | Macular thickness at the outer superior subfield (left) | 27816 |
|  |  | Macular thickness at the outer temporal subfield (left) | 27818 |
|  |  | Macular thickness at the outer inferior subfield (left) | 27812 |
|  |  | Macular thickness at the outer nasal subfield (left) | 27814 |
|  | The right-eyed dataset excludes the participants where the right parameter is the missing value | Macular thickness at the central subfield (right) | 27803 |
|  |  | Macular thickness at the inner superior subfield (right) | 27809 |
|  |  | Macular thickness at the inner temporal subfield (right) | 27811 |
|  |  | Macular thickness at the inner inferior subfield (right) | 27805 |
|  |  | Macular thickness at the inner nasal subfield (right) | 27807 |
|  |  | Macular thickness at the outer superior subfield (right) | 27817 |
|  |  | Macular thickness at the outer temporal subfield (right) | 27819 |
|  |  | Macular thickness at the outer inferior subfield (right) | 27813 |
|  |  | Macular thickness at the outer nasal subfield (right) | 27815 |
| Excluded low signal strength | Excluded image quality score less than 45 | QC - Image quality (left) | 28552 |
|  |  | QC - Image quality (right) | 28553 |
| Excluded low quality | Excluded 20% of the lowest ilm indicator value | QC - ILM indicator (left) | 28542 |
|  |  | QC - ILM indicator (right) | 28543 |
|  | Excluded 20% of the lowest validity count value | QC - Valid count (left) | 28544 |
|  |  | QC - Valid count (right) | 28545 |
|  | Excluded 20% of the lowest motion correlation value | QC - Min motion correlation (left) | 28546 |
|  |  | QC - Min motion correlation (right) | 28547 |
|  | Excluded the 20% with the highest motion delta value | QC - Max motion delta (left) | 28548 |
|  |  | QC - Max motion delta (right) | 28549 |
| Excluded refractive error >6D OR <-6D | refractive error = sphere power + 0.5 * cylinder power | Spherical power (left) | 5085 |
|  |  | Cylindrical power (left) | 5086 |
|  |  | Spherical power (right) | 5084 |
|  |  | Cylindrical power (right) | 5087 |
| Excluded vision worse than 0.1 logMAR | Excluded vision worse than 0.1 logMAR | logMAR, final (left) | 5208 |
|  |  | logMAR, final (right) | 5201 |
| exclude self-reported disorders | exclude self-reported ocular disorders(Except for eye surgery and corneal transplantation) | Tree-structured list used by clinic nurses to code non-cancer illness-eye trauma | 20002(1279) |
|  |  | Tree-structured list used by clinic nurses to code non-cancer illness-macular degeneration | 20002 (1528) |
|  |  | retinal detachment | 20002 (1281) |
|  |  | retinal artery/vein occlusion | 20002(1282) |
|  |  | cataract | 20002(1278) |
|  |  | retinal problem | 20002(1275) |
|  |  | diabetic eye disease | 20002(1276) |
|  | exclude self-reported glaucoma | glaucoma | 20002(1277) |
|  | exclude self-reported diabetes | diabetes | 20002(1220) |
|  |  | gestational diabetes | 20002(1221) |
|  |  | type 1 diabetes | 20002(1222) |
|  |  | type 2 diabetes | 20002(1223) |
| Excluded Eye surgery/corneal graft | Excluded Eye surgery | recent eye surgery | 20002(5181) |
|  | Excluded corneal graft | corneal graft | 20002(5328) |
| Excluded missing IOP, IOP >=22, IOP =< 5 | Both correction methods must be satisfied | Intra-ocular pressure, Goldmann-correlated (left) | 5263 |
|  |  | Intra-ocular pressure, Goldmann-correlated (right) | 5255 |
|  |  | Corneal hysteresis (left) | 5264 |
|  |  | Intra-ocular pressure, corneal-compensated (right) | 5254 |
| Excluded baseline dementia | One form of dementia prior to enrollment time was excluded | Date of attending assessment centre | 53 |
|  |  | Date F00 first reported (dementia in alzheimer's disease) | 130836 |
|  |  | Date F01 first reported (vascular dementia) | 130838 |
|  |  | Date F02 first reported (dementia in other diseases classified elsewhere) | 130840 |
|  |  | Date F03 first reported (unspecified dementia) | 130842 |

Abbreviation: IOP: intraocular pressure; ETDRS: Early treatment diabetic retinopathy study; LogMAR: Logarithm of the Minimum Angle of Resolution

**Supplementary Material 2. Primary analyses**

**SM 2 Table 1 - Primary cox regression models of Alzheimer’s disease**

| SD-OCT  measures | Model 1 | | Model 2 | | Model 3 | |
| --- | --- | --- | --- | --- | --- | --- |
|  | HR (95%CI) | *P* | HR (95%CI) | *P* | HR (95%CI) | *P* |
| Macular_Overall | 1.020 (1.007-1.032) | 0.002 | 1.007 (0.994-1.020) | 0.294 | 1.006 (0.993-1.019) | 0.369 |
| RNFL | 1.066 (1.023-1.111) | 0.002 | 1.026 (0.985-1.069) | 0.212 | 1.025 (0.983-1.069) | 0.253 |
| GC-IPL | 1.075 (1.044-1.107) | 0.000 | 1.036 (1.005-1.068) | 0.021 | 1.033 (1.001-1.066) | 0.044 |
| INL | 1.006 (0.937-1.080) | 0.865 | 0.976 (0.909-1.047) | 0.496 | 0.985 (0.915-1.061) | 0.698 |
| INL-RPE | 1.001 (0.980-1.022) | 0.939 | 0.996 (0.974-1.018) | 0.720 | 0.996 (0.974-1.018) | 0.706 |
| INL-ELM | 1.001 (0.975-1.028) | 0.943 | 1.005 (0.979-1.031) | 0.729 | 1.006 (0.979-1.033) | 0.684 |
| INL-RPE | 1.224 (1.068-1.403) | 0.004 | 0.968 (0.856-1.094) | 0.601 | 0.973 (0.860-1.099) | 0.656 |
| ISOS-RPE | 0.979 (0.937-1.022) | 0.331 | 0.972 (0.927-1.020) | 0.246 | 0.969 (0.925-1.016) | 0.195 |
| RPE | 1.046 (0.986-1.110) | 0.135 | 0.997 (0.935-1.063) | 0.927 | 0.997 (0.934-1.064) | 0.930 |
| RPE_CF | 1.014 (0.974-1.055) | 0.511 | 0.977 (0.935-1.022) | 0.310 | 0.984 (0.940-1.029) | 0.472 |
| RPE_SI | 1.017 (0.971-1.065) | 0.486 | 0.983 (0.935-1.034) | 0.517 | 0.985 (0.935-1.037) | 0.563 |
| RPE_TI | 0.991 (0.948-1.037) | 0.707 | 0.975 (0.929-1.024) | 0.313 | 0.978 (0.929-1.030) | 0.398 |
| RPE_II | 1.001 (0.957-1.046) | 0.981 | 0.967 (0.921-1.016) | 0.184 | 0.972 (0.924-1.023) | 0.278 |
| RPE_NI | 1.010 (0.966-1.057) | 0.648 | 0.993 (0.946-1.043) | 0.788 | 0.992 (0.943-1.043) | 0.743 |
| RPE_SO | 1.029 (0.962-1.100) | 0.405 | 0.994 (0.925-1.069) | 0.877 | 0.992 (0.922-1.068) | 0.837 |
| RPE_TO | 1.041 (0.983-1.103) | 0.167 | 1.020 (0.959-1.085) | 0.526 | 1.019 (0.957-1.086) | 0.552 |
| RPE_IO | 1.036 (0.965-1.112) | 0.335 | 0.978 (0.907-1.054) | 0.559 | 0.982 (0.910-1.060) | 0.641 |
| RPE_NO | 1.041 (0.986-1.098) | 0.148 | 1.011 (0.956-1.069) | 0.695 | 1.010 (0.954-1.070) | 0.728 |

Model 1: Unadjusted

Model 2: Adjusted for sociodemographic factors (age, sex, ethnicity, education, Townsend Deprivation index), smoking status

Model 3: Adjusted for Model 2 factors + disease-related risk factors (hypertension, hyperlipidemia, cardiovascular disease, and *APOE* ε4 status) + height + ocular factors (refractive error, intraocular pressure).

Abbreviation: HR, Hazard ratios; 95%CI, Confidential intervals; Macular_Overall, overall macular thickness; RNFL, retinal nerve fiber layer; GC-IPL, ganglion cell-inner plexiform layer; INL, inner nuclear layer; INL-RPE, inner nuclear layer-retinal pigment epithelial; INL-ELM, inner nuclear layer-external limiting membrane; ELM-ISOS, external limiting membrane-inner segment outer segment; ISOS-RPE, inner segment outer segment-retinal pigment epithelium; RPE, retinal pigment epithelium; CF, central subfield; SI, inner superior subfield; TI, inner temporal subfield; II, inner inferior subfield; NI, inner nasal subfield; SO, outer superior subfield; TO, outer temporal subfield; IO, outer inferior subfield; NO, outer nasal subfield.

**SM 2 Table 2 - Primary cox regression models of Frontotemporal dementia**

| SD-OCT  measures | Model 1 | | Model 2 | | Model 3 | |
| --- | --- | --- | --- | --- | --- | --- |
|  | HR (95%CI) | *P* | HR (95%CI) | *P* | HR (95%CI) | *P* |
| Macular_Overall | 1.014 (0.961-1.070) | 0.613 | 1.007 (0.954-1.064) | 0.795 | 1.010 (0.955-1.069) | 0.722 |
| RNFL | 1.150 (0.957-1.383) | 0.136 | 1.113 (0.923-1.341) | 0.262 | 1.102 (0.911-1.334) | 0.315 |
| GC-IPL | 1.024 (0.902-1.164) | 0.712 | 1.006 (0.885-1.143) | 0.932 | 1.017 (0.892-1.159) | 0.806 |
| INL | 1.140 (0.833-1.560) | 0.413 | 1.163 (0.848-1.594) | 0.350 | 1.216 (0.874-1.691) | 0.246 |
| INL-RPE | 0.980 (0.895-1.073) | 0.661 | 0.977 (0.890-1.071) | 0.617 | 0.979 (0.892-1.075) | 0.661 |
| INL-ELM | 1.008 (0.900-1.128) | 0.894 | 1.014 (0.906-1.135) | 0.807 | 1.024 (0.912-1.150) | 0.688 |
| INL-RPE | 0.896 (0.568-1.415) | 0.638 | 0.809 (0.527-1.241) | 0.331 | 0.782 (0.502-1.220) | 0.278 |
| ISOS-RPE | 0.910 (0.745-1.112) | 0.357 | 0.889 (0.720-1.097) | 0.272 | 0.880 (0.713-1.086) | 0.233 |
| RPE | 1.152 (0.861-1.541) | 0.340 | 1.153 (0.853-1.558) | 0.353 | 1.175 (0.868-1.590) | 0.296 |
| RPE_CF | 1.211 (0.984-1.491) | 0.070 | 1.204 (0.977-1.483) | 0.082 | 1.233 (0.993-1.531) | 0.058 |
| RPE_SI | 1.386 (1.037-1.852) | 0.027 | 1.386 (1.039-1.849) | 0.026 | 1.409 (1.060-1.871) | 0.018 |
| RPE_TI | 1.121 (0.889-1.414) | 0.335 | 1.156 (0.904-1.478) | 0.248 | 1.171 (0.910-1.507) | 0.220 |
| RPE_II | 1.238 (0.955-1.606) | 0.108 | 1.233 (0.962-1.581) | 0.099 | 1.255 (0.983-1.603) | 0.068 |
| RPE_NI | 1.049 (0.857-1.285) | 0.642 | 1.063 (0.863-1.308) | 0.567 | 1.076 (0.870-1.332) | 0.499 |
| RPE_SO | 1.373 (0.898-2.101) | 0.143 | 1.436 (0.932-2.214) | 0.101 | 1.442 (0.928-2.241) | 0.104 |
| RPE_TO | 1.056 (0.819-1.361) | 0.674 | 1.098 (0.841-1.434) | 0.492 | 1.116 (0.844-1.475) | 0.440 |
| RPE_IO | 1.375 (0.898-2.104) | 0.143 | 1.384 (0.914-2.093) | 0.124 | 1.442 (0.937-2.218) | 0.096 |
| RPE_NO | 1.221 (0.922-1.618) | 0.164 | 1.261 (0.951-1.673) | 0.108 | 1.260 (0.946-1.676) | 0.114 |

Model 1: Unadjusted

Model 2: Adjusted for sociodemographic factors (age, sex, ethnicity, education, Townsend Deprivation index), smoking status

Model 3: Adjusted for Model 2 factors + disease-related risk factors (hypertension, hyperlipidemia, cardiovascular disease, and *APOE* ε4 status) + height + ocular factors (refractive error, intraocular pressure).

Abbreviation: HR, Hazard ratios; 95%CI, Confidential intervals; Macular_Overall, overall macular thickness; RNFL, retinal nerve fiber layer; GC-IPL, ganglion cell-inner plexiform layer; INL, inner nuclear layer; INL-RPE, inner nuclear layer-retinal pigment epithelial; INL-ELM, inner nuclear layer-external limiting membrane; ELM-ISOS, external limiting membrane-inner segment outer segment; ISOS-RPE, inner segment outer segment-retinal pigment epithelium; RPE, retinal pigment epithelium; CF, central subfield; SI, inner superior subfield; TI, inner temporal subfield; II, inner inferior subfield; NI, inner nasal subfield; SO, outer superior subfield; TO, outer temporal subfield; IO, outer inferior subfield; NO, outer nasal subfield.

**Supplementary Material 3. Nonlinear test and violation of the proportional hazard assumptions**

**SM 3 Figure 1 - Restricted cubic spline curves**

**
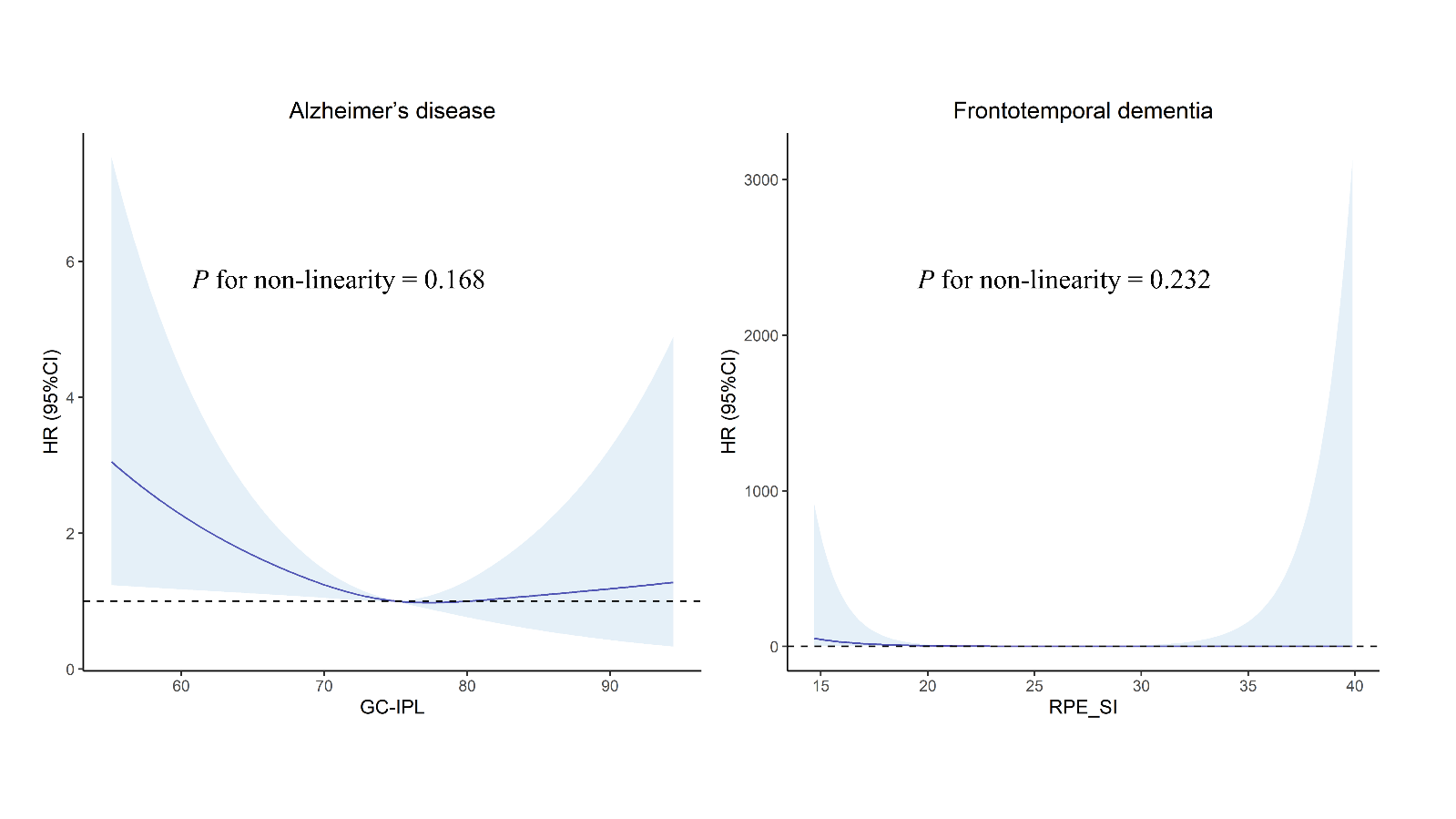
**

**SM 3 Figure 2 - Schoenfeld's residuals test for Cox proportional hazard regression models**

**
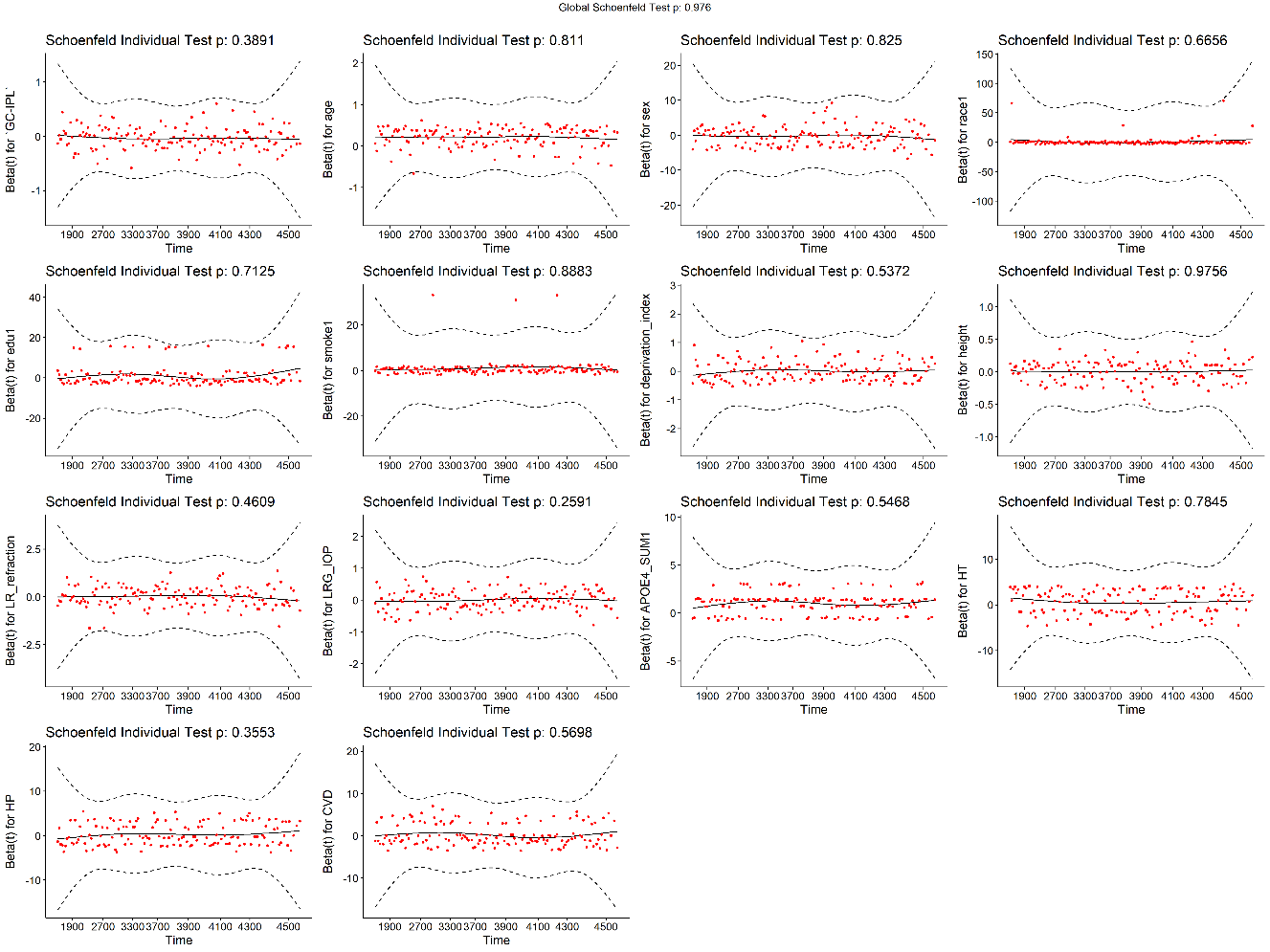

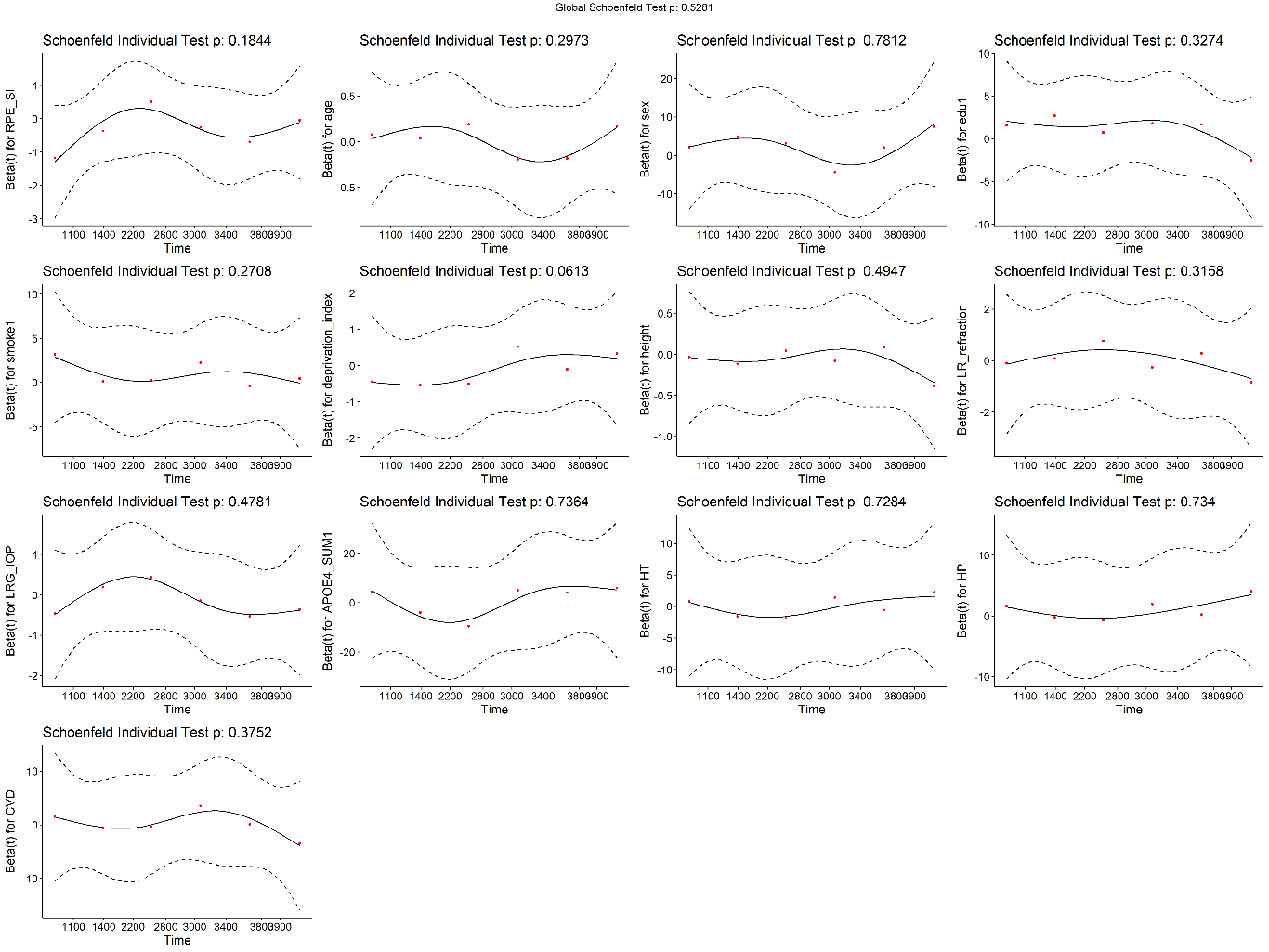
**

**Supplementary Material 4. Subgroup analyses**

**SM 4 Table 1 - Association Between GC-IPL and Alzheimer’s disease in subgroups**

| Outcome | Subgroup | GC-IPL | | | | | | | |
| --- | --- | --- | --- | --- | --- | --- | --- | --- | --- |
|  |  | Model 1 | | Model 2 | | Model 3 | | | |
|  |  | HR (95%CI) | *P* | HR (95%CI) | *P* | HR (95%CI) | *P* | | |
| Alzheimer’s  Disease | Age≤55  (incidents= 7) | 0.916 (0.796-1.053) | 0.217 | 0.902 (0.786-1.034) | 0.139 | 0.908 (0.786-1.049) | | 0.188 |  |
|  | Age >55  (incidents=140) | 1.055 (1.024-1.088) | 0.000 | 1.043 (1.012-1.076) | 0.007 | 1.039 (1.006-1.073) | | 0.019 |  |
|  | Male  (incidents=70) | 1.107 (1.062-1.153) | 0.000 | 1.064 (1.020-1.111) | 0.004 | 1.057 (1.011-1.105) | | 0.014 |  |
|  | Female  (incidents=77) | 1.043 (1.000-1.087) | 0.049 | 1.007 (0.965-1.051) | 0.753 | 1.007 (0.962-1.053) | | 0.774 |  |
|  | With CVD  (incidents=41) | 1.090 (1.031-1.153) | 0.003 | 1.078 (1.017-1.142) | 0.011 | 1.087 (1.023-1.155) | | 0.007 |  |
|  | Without CVD  (incidents=106) | 1.062 (1.026-1.100) | 0.001 | 1.022 (0.986-1.059) | 0.232 | 1.012 (0.975-1.050) | | 0.534 |  |

Model 1: Unadjusted

Model 2: Adjusted for sociodemographic factors (age, sex, ethnicity, education, Townsend Deprivation index), smoking status

Model 3: Adjusted for Model 2 factors + disease-related risk factors (hypertension, hyperlipidemia, cardiovascular disease, and *APOE* ε4 status) + height + ocular factors (refractive error, intraocular pressure).

Abbreviation: HR, Hazard ratios; 95%CI, Confidential intervals; CVD, Cardiovascular disease; GC-IPL, ganglion cell-inner plexiform layer.

**SM 4 Table 2 - Association Between RPE_SI and Frontotemporal dementia in subgroups**

| Outcome | Subgroup | RPE_SI | | | | | |
| --- | --- | --- | --- | --- | --- | --- | --- |
|  |  | Model 1 | | Model 2 | | Model 3 | |
|  |  | HR (95%CI) | *P* | HR (95%CI) | *P* | HR (95%CI) | *P* |
| Frontotemporal  dementia | Age≤55  (incidents=2) | 1.560 (0.898-2.710) | 0.114 | 1.630 (0.879-3.023) | 0.121 | 2.042 (1.021-4.083) | 0.043 |
|  | Age >55  (incidents=4) | 1.307 (0.926-1.846) | 0.128 | 1.358 (0.973-1.895) | 0.072 | 1.355 (0.984-1.865) | 0.062 |
|  | Male  (incidents=5) | 1.390 (1.018-1.898) | 0.038 | 1.444 (1.051-1.982) | 0.023 | 1.465 (1.067-2.012) | 0.018 |
|  | Female  (incidents=1) | 1.195 (0.638-2.238) | 0.579 | 1.220 (0.616-2.415) | 0.569 | 1.784 (1.000-Inf) | 1.000 |
|  | With CVD  (incidents=2) | 2.097 (1.174-3.747) | 0.012 | 2.511 (0.943-6.691) | 0.066 | / | 0.990 |
|  | Without CVD  (incidents=4) | 1.180 (0.873-1.596) | 0.281 | 1.180 (0.877-1.588) | 0.274 | 1.191 (0.897-1.583) | 0.227 |

Model 1: Unadjusted

Model 2: Adjusted for sociodemographic factors (age, sex, ethnicity, education, Townsend Deprivation index), smoking status

Model 3: Adjusted for Model 2 factors + disease-related risk factors (hypertension, hyperlipidemia, cardiovascular disease, and *APOE* ε4 status) + height + ocular factors (refractive error, intraocular pressure).

Abbreviation: HR, Hazard ratios; 95%CI, Confidential intervals; CVD, Cardiovascular disease; RPE_ SI, retinal pigment epithelium at inner superior subfield.

**Supplementary Material 5. Sensitivity analyses**

**SM 5 Table 1 - Follow-up-restricted models**

| Exposure | Outcome | Follow-up-restricted models | | | | | |
| --- | --- | --- | --- | --- | --- | --- | --- |
|  |  | Model 1 | | Model 2 | | Model 3 | |
|  |  | HR (95%CI) | *P* | HR (95%CI) | *P* | HR (95%CI) | *P* |
| GC-IPL | Alzheimer’s  Disease  (incidents= 140) | 1.078 (1.046-1.111) | 0.000 | 1.039 (1.008-1.072) | 0.014 | 1.035 (1.003-1.069) | 0.034 |
| RPE_SI | Frontotemporal  Dementia  (incidents= 6) | 1.129 (0.849-1.502) | 0.402 | 1.127 (0.851-1.492) | 0.403 | 1.154 (0.867-1.535) | 0.326 |

Model 1: Unadjusted

Model 2: Adjusted for sociodemographic factors (age, sex, ethnicity, education, Townsend Deprivation index), smoking status

Model 3: Adjusted for Model 2 factors + disease-related risk factors (hypertension, hyperlipidemia, cardiovascular disease, and *APOE* ε4 status) + height + ocular factors (refractive error, intraocular pressure).

Abbreviation: HR, Hazard ratios; 95%CI, Confidential intervals; GC-IPL, ganglion cell-inner plexiform layer; RPE_ SI, retinal pigment epithelium at inner superior subfield.

**SM 5 Table 2 - *APOE* ε4-restricted models**

| Exposure | Outcome | *APOE* ε4-restricted models | | | | | |
| --- | --- | --- | --- | --- | --- | --- | --- |
|  |  | Model 1 | | Model 2 | | Model 3 | |
|  |  | HR (95%CI) | *P* | HR (95%CI) | *P* | HR (95%CI) | *P* |
| GC-IPL | Alzheimer’s  Disease  (incidents= 34) | 1.114 (1.049-1.184) | 0.000 | 1.076 (1.012-1.144) | 0.020 | 1.064 (0.998-1.134) | 0.060 |
| RPE_SI | Frontotemporal  Dementia  (incidents= 5) | 1.743 (1.168-2.600) | 0.007 | 1.785 (1.164-2.738) | 0.008 | 1.772 (1.161-2.706) | 0.008 |

Model 1: Unadjusted

Model 2: Adjusted for sociodemographic factors (age, sex, ethnicity, education, Townsend Deprivation index), smoking status

Model 3: Adjusted for Model 2 factors + disease-related risk factors (hypertension, hyperlipidemia, cardiovascular disease, and *APOE* ε4 status) + height + ocular factors (refractive error, intraocular pressure).

Abbreviation: HR, Hazard ratios; 95%CI, Confidential intervals; GC-IPL, ganglion cell-inner plexiform layer; RPE_ SI, retinal pigment epithelium at inner superior subfield; *APOE* ε4, Apolipoprotein E4.

**Supplementary Material 6. Age- and sex- matched cohort analyses**

**SM 6 Table 1 - Baseline characteristics of study participants by incident AD status**

| Characteristics | All participants  (N=17261) | No Incident AD  (N =17113) | Incident AD  (N =148) | *P* |
| --- | --- | --- | --- | --- |
| Age | 61.24(4.49) | 61.21(4.48) | 64.36(3.93) | <0.001*** |
| Male (%) | 8947(51.8) | 8870(51.8) | 77(52.0) | 1 |
| Race, No. (%) |  |  |  | 0.971‡ |
| White | 16363(94.8) | 16221(94.8) | 142(95.9) |  |
| mixed/other ethnic | 244(1.4) | 242(1.4) | 2(1.4) |  |
| Asian/Indian | 278(1.6) | 276(1.6) | 2(1.4) |  |
| Black | 234(1.4) | 232(1.4) | 2(1.4) |  |
| Chinese | 35(0.2) | 35(0.2) | 0(0.0) |  |
| Education, No. (%) |  |  |  | 0.006** |
| Low | 1047(6.1) | 1032(6.0) | 15(10.1) |  |
| Moderate | 4455(25.8) | 4422(25.8) | 33(22.3) |  |
| High | 8615(49.9) | 8554(50.0) | 61(41.2) |  |
| Laterality=right eye | 7848(45.5) | 7779(45.5) | 69(46.6) | 0.841 |
| BMI, kg/m^2^ | 27.19(4.38) | 27.19(4.38) | 26.77(4.35) | 0.244 |
| Smoking status (%) |  |  |  | 0.039* |
| Never | 9005(52.2) | 8935(52.2) | 70(47.3) |  |
| Previous | 6822(39.5) | 6757(39.5) | 65(43.9) |  |
| Current | 1346(7.8) | 1336(7.8) | 10(6.8) |  |
| Alcohol status (%) |  |  |  | 0.393 |
| Never | 652(3.8) | 645(3.8) | 7(4.7) |  |
| Previous | 550(3.2) | 542(3.2) | 8(5.4) |  |
| Current | 16006(92.9) | 15873(92.9) | 133(89.9) |  |
| Height(cm) | 168.64(9.17) | 168.65(9.16) | 167.98(9.54) | 0.379 |
| Townsend deprivation index | -1.44(2.79) | -1.44(2.79) | -1.49(2.64) | 0.83 |
| Visual acuity, logMAR | -0.06(0.09) | -0.06(0.09) | -0.04(0.08) | 0.04* |
| Refractive error | 0.27(1.93) | 0.27(1.93) | 0.62(1.84) | 0.026* |
| Intraocular pressure | 15.38(3.06) | 15.38(3.07) | 15.49(2.84) | 0.656 |
| Hypertension (%) | 5979(34.6) | 5890(34.4) | 89(60.1) | <0.001*** |
| Hyperlipidemia (%) | 3436(19.9) | 3380(19.8) | 56(37.8) | <0.001*** |
| Cardiovascular disease (%) | 2687(15.6) | 2645(15.5) | 42(28.4) | <0.001*** |
| Ischemic heart disease (%) | 2290(13.3) | 2254(13.2) | 36(24.3) | <0.001*** |
| Stroke (%) | 484(2.8) | 476(2.8) | 8(5.4) | 0.094 |
| *APOE* ε4 (%) |  |  |  | <0.001*** |
| 0 allele | 10096(58.5) | 10062(58.8) | 34(23.0) |  |
| 1 allele | 3717(21.5) | 3653(21.3) | 64(43.2) |  |
| 2 allele | 345(2.0) | 325(1.9) | 20(13.5) |  |
| Overall macular thickness | 277.19(13.06) | 277.21(13.06) | 275.17(12.89) | 0.059 |
| RNFL | 28.13(4.11) | 28.13(4.11) | 27.48(4.26) | 0.052 |
| GC-IPL | 74.27(5.49) | 74.28(5.48) | 72.90(5.85) | 0.002** |
| INL | 32.57(2.29) | 32.57(2.29) | 32.65(2.42) | 0.654 |
| INL-RPE | 142.22(7.51) | 142.22(7.51) | 142.14(6.69) | 0.898 |
| RPE | 25.05(2.62) | 25.05(2.62) | 25.04(2.30) | 0.971 |

Data are presented as mean (SD), median (IQR), or n (%). *P* values were calculated using t-test for continuous variables or Chi-square test for categorical variables. ‡Use Fisher’s Exact Test. ***indicates a *P* value <0.001. **indicates a *P* value <0.01. *indicates a *P* value <0.05.

Abbreviations: BMI, body mass index; *APOE* ε4, Apolipoprotein E4; RNFL, retinal nerve fiber layer; GC-IPL, ganglion cell-inner plexiform layer; INL, inner nuclear layer; INL-RPE, inner nuclear layer-retinal pigment epithelial; RPE, retinal pigment epithelium.

**SM 6 Table 2 - Baseline characteristics of study participants by incident FTD status**

| Characteristics | All participants  (N=4709) | No Incident FTD  (N =4701) | Incident FTD  (N =8) | *P* |
| --- | --- | --- | --- | --- |
| Age | 60.90(6.05) | 60.90(6.05) | 60.38(7.33) | 0.806 |
| Male (%) | 2111(44.8) | 2108(44.8) | 3(37.5) | 0.951 |
| Race, No. (%) |  |  |  | 0.41‡ |
| White | 4460(94.7) | 4453(94.7) | 7(87.5) |  |
| mixed/other ethnic | 77(1.6) | 76(1.6) | 1(12.5) |  |
| Asian/Indian | 72(1.5) | 72(1.5) | 0(0.0) |  |
| Black | 61(1.3) | 61(1.3) | 0(0.0) |  |
| Chinese | 9(0.2) | 9(0.2) | 0(0.0) |  |
| Education, No. (%) |  |  |  | 0.108 |
| Low | 291(6.2) | 291(6.2) | 0(0.0) |  |
| Moderate | 1238(26.3) | 1234(26.2) | 4(50.0) |  |
| High | 2325(49.4) | 2324(49.4) | 1(12.5) |  |
| Laterality=right eye | 2211(47.0) | 2209(47.0) | 2(25.0) | 0.737 |
| BMI, kg/m^2^ | 27.26(4.41) | 27.26(4.41) | 25.86(4.63) | 0.371 |
| Smoking status (%) |  |  |  | 0.349 |
| Never | 2375(50.4) | 2371(50.4) | 4(50.0) |  |
| Previous | 1929(41.0) | 1927(41.0) | 2(25.0) |  |
| Current | 386(8.2) | 384(8.2) | 2(25.0) |  |
| Alcohol status (%) |  |  |  | 0.906 |
| Never | 156(3.3) | 156(3.3) | 0(0.0) |  |
| Previous | 146(3.1) | 146(3.1) | 0(0.0) |  |
| Current | 4398(93.5) | 4390(93.5) | 8(100.0) |  |
| Height(cm) | 169.55(9.04) | 169.55(9.05) | 169.19(7.12) | 0.909 |
| Townsend deprivation index | -1.45(2.79) | -1.45(2.79) | -1.57(3.12) | 0.905 |
| Visual acuity, logMAR | -0.06(0.09) | -0.06(0.09) | -0.05(0.09) | 0.821 |
| Refractive error | 0.30(1.90) | 0.30(1.90) | 0.73(1.67) | 0.52 |
| Intraocular pressure | 15.38(3.07) | 15.38(3.07) | 14.22(2.97) | 0.285 |
| Hypertension (%) | 1654(35.1) | 1651(35.1) | 3(37.5) | 1 |
| Hyperlipidemia (%) | 974(20.7) | 971(20.7) | 3(37.5) | 0.46 |
| Cardiovascular disease (%) | 786(16.7) | 784(16.7) | 2(25.0) | 0.876 |
| Ischemic heart disease (%) | 665(14.1) | 663(14.1) | 2(25.0) | 0.707 |
| Stroke (%) | 145(3.1) | 144(3.1) | 1(12.5) | 0.603 |
| *APOE* ε4 (%) |  |  |  | 0.953 |
| 0 allele | 2767(58.8) | 2762(58.8) | 5(62.5) |  |
| 1 allele | 1010(21.4) | 1008(21.4) | 2(25.0) |  |
| 2 allele | 75(1.6) | 75(1.6) | 0(0.0) |  |
| Overall macular thickness | 277.51(12.87) | 277.51(12.87) | 276.06(9.83) | 0.751 |
| RNFL | 28.17(4.09) | 28.17(4.09) | 26.36(3.56) | 0.212 |
| GC-IPL | 74.35(5.47) | 74.35(5.47) | 74.31(4.10) | 0.983 |
| INL | 32.65(2.31) | 32.65(2.31) | 32.02(1.87) | 0.446 |
| INL-RPE | 142.34(7.44) | 142.34(7.44) | 143.36(3.74) | 0.696 |
| RPE | 25.14(2.66) | 25.14(2.66) | 24.41(2.98) | 0.442 |

Data are presented as mean (SD), median (IQR), or n (%). *P* values were calculated using t-test for continuous variables or Chi-square test for categorical variables. ‡Use Fisher’s Exact Test. ***indicates a *P* value <0.001. **indicates a *P* value <0.01. *indicates a *P* value <0.05.

Abbreviations: BMI, body mass index; *APOE* ε4, Apolipoprotein E4; RNFL, retinal nerve fiber layer; GC-IPL, ganglion cell-inner plexiform layer; INL, inner nuclear layer; INL-RPE, inner nuclear layer-retinal pigment epithelial; RPE, retinal pigment epithelium.

**SM 6 Figure 1 - Associations of OCT parameters and incident AD/FTD**

**
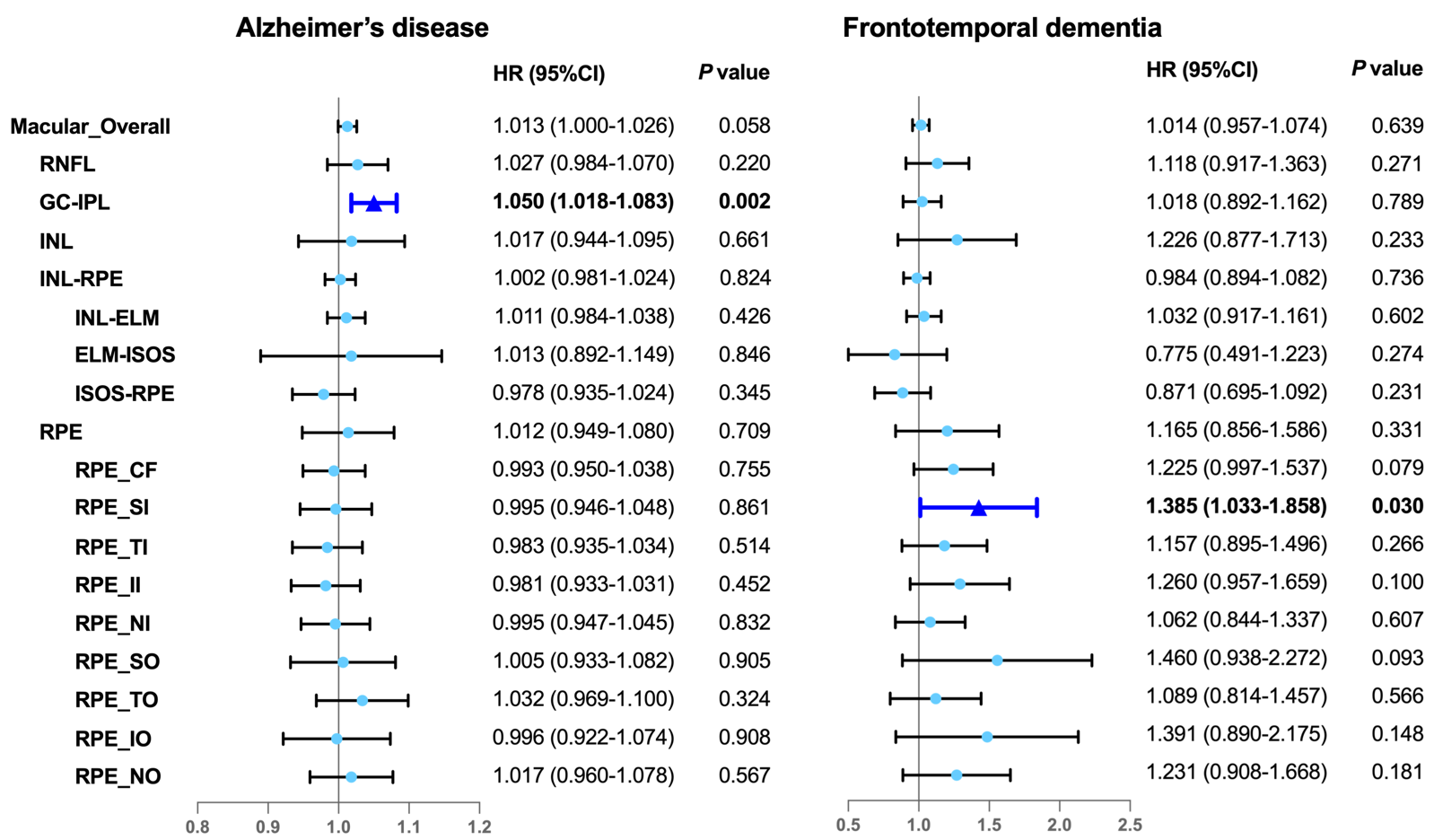
**

The solid color box stands for statistically significant associations (p < 0.05), whereas the lighter color box indicates null associations (p > 0.05). Model adjusted sociodemographic factors (ethnicity, education, Townsend Deprivation index), smoking status, disease-related risk factors (hypertension, hyperlipidemia, cardiovascular disease, and *APOE ε4* status), height and ocular factors (refractive error, intraocular pressure).

Abbreviations: HR, hazard ratio; 95% CI, 95% confidence interval; RNFL, retinal nerve fiber layer; GC-IPL, ganglion cell-inner plexiform layer; INL, inner nuclear layer; INL-RPE, inner nuclear layer-retinal pigment epithelial; INL-ELM, inner nuclear layer-external limiting membrane; ELM-ISOS, external limiting membrane-inner segment outer segment; ISOS-RPE, inner segment outer segment-retinal pigment epithelium; RPE, retinal pigment epithelium; CF, central subfield; SI, inner superior subfield; TI, inner temporal subfield; II, inner inferior subfield; NI, inner nasal subfield; SO, outer superior subfield; TO, outer temporal subfield; IO, outer inferior subfield; NO, outer nasal subfield.

**Appendix 1 - UK Biobank codes for dementia diagnosis and classification**

| **Diagnosis** | **Code Type** | **Codes** |
| --- | --- | --- |
| All-cause dementia | ICD-9 | 331.0, 290.4, 331.1, 290.2, 290.3, 291.2, 294.1, 331.2, 331.5 |
|  | ICD-10 | F00, F00.0, F00.1, F00.2, F00.9, G30, G30.0, G30.1, G30.8, G30.9, F01, F01.0, F01.1, F01.2, F01.3, F01.8, F01.9, I67.3, F02.0, G31.0, A81.0, F02, F02.1, F02.2, F02.3, F02.4, F02.8, F03, F05.1, F10.6, G31.1, G31.8 |
| Alzheimer’s disease | ICD-9 | 331.0 |
|  | ICD-10 | F00, F00.0, F00.1, F00.2, F00.9, G30, G30.0, G30.1, G30.8, G30.9 |
| Vascular Dementia | ICD-9 | 290.4 |
|  | ICD-10 | F01, F01.0, F01.1, F01.2, F01.3, F01.8, F01.9 |
| Frontotemporal Dementia | ICD-9 | 331.1 |
|  | ICD-10 | F02.0, G31.0 |
|  | | |

**Appendix 2 - Information of missing data**

|  | |
| --- | --- |
| **Variates** | **No. of sample with missing covariates** |
| Age | 0 |
| Sex | 0 |
| Ethnicity | 66 |
| Education | 3897 |
| Townsend Deprivation index | 43 |
| Smoking status | 170 |
| Hypertension | 0 |
| Hyperlipidemia | 0 |
| Cardiovascular disease | 0 |
| Height | 95 |
| Refractive error | 0 |
| Intraocular pressure | 0 |
| *APOE* ɛ4 | 5320 |
| * Table represents the sample containing missing data of covariates based on 30573 participants.  Abbreviation: *APOE* ε4, Apolipoprotein E4 | |
